# Recovering parasites from mummies and coprolites: an epidemiological approach

**DOI:** 10.1186/s13071-018-2729-4

**Published:** 2018-04-16

**Authors:** Morgana Camacho, Adauto Araújo, Johnica Morrow, Jane Buikstra, Karl Reinhard

**Affiliations:** 1 Escola Nacional de Saúde Pública Sergio Arouca/Fundação Oswaldo Cruz (ENSP/FIOCRUZ), Rua Leopoldo Bulhões, 1480, Manguinhos, Rio de Janeiro, RJ 21041-210 Brazil; 2 Department of Physical & Life Sciences, Chadron State College, 1000 Main Street, Chadron, NE 69337 USA; 3 School of Evolution and Social Change, Arizona State University, Tempe, AZ USA; 4 Pathoecology Laboratory, School of Natural Resources, University of Nebraska – Lincoln, Lincoln, NE 68583-0987 USA

**Keywords:** Coprolite, Quantification, Epidemiology, Overdispersion, Parasite

## Abstract

In the field of archaeological parasitology, researchers have long documented the distribution of parasites in archaeological time and space through the analysis of coprolites and human remains. This area of research defined the origin and migration of parasites through presence/absence studies. By the end of the 20th century, the field of pathoecology had emerged as researchers developed an interest in the ancient ecology of parasite transmission. Supporting studies were conducted to establish the relationships between parasites and humans, including cultural, subsistence, and ecological reconstructions. Parasite prevalence data were collected to infer the impact of parasitism on human health. In the last few decades, a paleoepidemiological approach has emerged with a focus on applying statistical techniques for quantification. The application of egg per gram (EPG) quantification methods provide data about parasites’ prevalence in ancient populations and also identify the pathological potential that parasitism presented in different time periods and geographic places. Herein, we compare the methods used in several laboratories for reporting parasite prevalence and EPG quantification. We present newer quantification methods to explore patterns of parasite overdispersion among ancient people. These new methods will be able to produce more realistic measures of parasite infections among people of the past. These measures allow researchers to compare epidemiological patterns in both ancient and modern populations.

## Background

Parasite evidence has been recovered from mummies, coprolites and skeletons for six decades. During this time, parasitology as applied to archaeology has become increasingly quantitative. As detailed in several reviews [1–3], the focus on quantification developed as research goals changed. In turn, new research perspectives were envisioned as methods were refined. Today, we are at a point at which parasitological data have distinct relevance to paleopathologists. Herein, we review the methods and accumulated data sets to address various historical goals and new potentials for the field.

Between 1955 and 1969, pioneering researchers developed methods for the recovery of parasite evidence and published their findings for several regions [1–3]. This approach reached its most successful year in 1969 with the publication of three articles in *Science*, one reporting the oldest pinworm [4], another reporting the oldest thorny-headed worm [5] and the third reporting no evidence of infection in 50 examined samples [6]. The latter paper was especially noteworthy for recognizing the significance of negative data in comparing the relative infection state between archaeological cultures. That theme would be further developed during the 1980s. Subsequent to 1969, the interest among archaeologists and parasitologists led to expanded analysis and new research goals.

In the decade of the 1970s, the analysis of large numbers of coprolites archived in museums intensified (Table 1). From these collections, parasite prevalence was assessed [1–3]. In modern parasitology, prevalence is a statistical concept referring to the number of cases of an infection  disease that are present in a particular population at a given time. This has to be carefully approached archaeologically because the actual population represented by the coprolite series has to be assessed by field and museum sampling. This led to provenience-based sampling strategies in the field and laboratory. The development of sampling methods is discussed below in Development of methods. A landmark book, tracing parasitism and diet adaptations across the Great Basin and adjoining the Colorado Plateau, was published in 1977 by Gary Fry [7]. This overview was based on prevalence quantification and details stratigraphic sampling of coprolite samples. The book, *Analysis of Prehistoric Coprolites from Utah*, defined the biogeography of parasite infection for this region through time and subsistence strategies.

**Table 1:** Studies on coprolites and mummies published from 1944 through 2016

Provenience	Material	*n* ^a^	Type^b^	Method	Year/Reference
Drobnitz Girl, Germany	mummy	1	A	?	1944 [75]
Karwinden Man, Poland	mummy	1	A	?	1944 [75]
El Plomo, Chile	coprolite/mummy	1	A	?	1954 [76]
Ming Dynasty, Guangzhou, China	coprolite/ mummy	1	A	?	1956 [17]
Grauballe Man, Denmark	mummy	1	A	?	1958 [17]
Tollund Man, Denmark	mummy	1	A	?	1958 [77]
Odra River, Poland	coprolites	?	?	?	1960 [78]
Nahal-Mishmar Valley, Israel	coprolites	2	A	?	1961 [79]
Step House, CO, USA	coprolites	20	AB	Callen	1965 [43]
Lovelock Cave, CA, USA	coprolites	168	AB	Callen	1970 [9]
Frightful Cave, Mexico	coprolites	32	AB	Callen	1970 [9]
Clyde’s Cavern, USA	coprolites	16	AB	Callen	1971 [9]
Lion House, CO, USA	coprolites	4	A	Callen	1972 [9]
Hoy House, CO, USA	coprolites	56	AB	Callen	1972 [9]
Western Han Dynasty, Changsha City, China	coprolite/mummy	1	A	?	1973 [17]
Pisco, Peru	mummy intestine section	1	A	Callen	1974 [80]
Upper Salts Cave, KY, USA	coprolites	8	A	Callen	1974 [81]
Ch'angsha, Hunan Province, China	mummy intestine section	1	A	?	1974 [82]
Glenn Canyon, USA	coprolites	40	AB	Callen	1977 [9]
Danger Cave, UT, USA	coprolites	46	AB	Callen	1977 [9]
Hogup Cave, UT, USA	coprolites	60	AB	Callen	1977 [9]
Hinds Cave, TX USA	coprolites	13	A	Callen	1978 [9]
Canyon del Muerto, NM, USA	coprolites/ mummies	2	A	Callen	1980 [83]
Gentio II, MS, Brazil	coprolites	22	AB	Lutz	1980 [84]
Itacambira, MG, Brazil	coprolites/ mummies	3	A	Lutz	1981 [85]
Han Dynasty Jinagling City, China	coprolite/mummy	1	A	?	1981 [17]
Boqueirão Soberbo, MS, Brazil	coprolites	?	?	Lutz	1982 [86]
Gentio II, MS, Brazil	coprolite/mummy	1	A	Lutz	1983 [87]
Llods Street Pavement, UK	coprolite	1	A	HCl	1983 [59]
Los Gavilanes, Peru	coprolites	52	AB	?	1983 [88]
Hinds Cave, TX, USA	coprolites	7	A	Callen	1983 [9]
Tiliviche, Chile	coprolites	26	AB	Lutz	1984 [89]
Chu Dynasty, Jingmen City, South Korea	coprolite/mummy	1	A	?	1984 [17]
Caserones, Chile	coprolites	10	A	Lutz	1985 [90]
Antelope House, AZ, USA	coprolites	90	AB	Callen	1986 [9]
Lindow Man, England	mummy	1	A	?	1986 [75]
Chaco Canyon, NM, USA	coprolites	20	AB	Callen	1987 [9]
Turkey Pen Cave, UT, USA	coprolites	24	AB	Callen	1987 [9]
Antelope House, AZ, USA	coprolites	62	AB	Callen	1987 [9]
Dust Devil Cave, UT, USA	coprolites	100	AB	Callen	1987 [9]
Salmon Ruins, NM, USA	coprolites	112	AB	Callen	1987 [9]
Pedra Furada, PI, Brazil	coprolites	17	AB	Lutz	1987 [91]
Bighorn Sheep Ruin, UT, USA	coprolites	20	AB	Callen?	1988 [9]
Qilaleitsoq, Greenland	coprolite/mummy	1	A	?	1989 [92]
Big Bone Cave	coprolites	8	A	Formalin- ethyl acetate sedimention	1989 [93]
Estrago Cave, PE, Brazil	coprolites	4	A	Lutz	1989 [94]
NAN Ranch Ruin, NM, USA	coprolites/burial	1	A	Callen/Lyco	1989 [58]
Ventana Cave, AZ, USA	coprolite/mummy	1	A	Callen/Lyco	1991 [95]
Antelope House, AZ, USA	coprolites	180	AB	Callen	1992 [9]
Inscription House AZ, USA	coprolites	16	AB	Callen	1992 [9]
Baker Cave, TX, USA	coprolites	17	AB	Callen/Lyco	1992 [9]
Hinds Cave, TX, USA	coprolites	39	AB	Callen/Lyco	1992 [9]
Dan Canyon, AZ, USA	coprolites/burial	1	A	Callen/Lyco	1992 [96]
Klethla, AZ, USA	sediments/burial	1	A	Chemical	1992 [9]
Montbéliard, France	coprolites/sediments	?	?	Reims	2002 [97]
León, Spain	mummy contents	4	A	Flotation	2003 [57]
Bighorn Cave, AZ, USA	coprolites	35	ABCD	Callen/Lyco	2002 [98]
Chiribaya, Peru	coprolites	29	AB	Callen	2003 [99]
Chiribaya, Peru	coprolites/mummy	43	ABCD	Callen/Lyco	2003 [10]
Skyles Mummy, TX, USA	coprolites/mummy	1	AC	Callen/Lyco	2003 [100]
Lluta Valley, Chile	coprolites/mummy	39	ABCD	Callen/Lyco	2003 [50]
Diverse sites, Switzerland and Germany	coprolites and sediments	89	AB	Reims	2005 [101]
Lapa do Boquete, Brazil	coprolite/mummy	1	AC	Callen/Lyco	2002 [51]
**Gangneung, South Korea**	coprolite/mummy	1	AB	Lutz	2007 [39]
**Yangju, South Korea**	coprolite/mummy	1	AB	Lutz	2007 [39]
**SN2-19-1, South Korea**	coprolite/ mummy	1	AB	Lutz	2007 [39]
**SN1-2, South Korea**	coprolite/ mummy	1	AB	Lutz	2007 [39]
**SN3-7-1, South Korea**	coprolite/ mummy	1	AB	Lutz	2007 [39]
**SN2-19-2, South Korea**	coprolite/ mummy	1	AB	Lutz	2007 [39]
**Hadong-1, South Korea**	coprolite/mummy	1	AB	Lutz	2008 [39]
**GJ1-2, South Korea**	coprolite/ mummy	1	AB	Lutz	2008 [39]
**Yongin, South Korea**	coprolite/mummy	1	AB	Lutz	2009 [39]
**Waegwan, South Korea**	coprolite/ mummy	1	AB	Lutz	2010 [39]
**Seocheon, South Korea**	coprolite/ mummy	1	AB	Lutz	2010 [39]
**Sinnae, South Korea**	coprolite/ mummy	1	AB	Lutz	2010 [39]
Chinchorro, Chile	coprolite/mummy	24	ABCD	Callen/Lyco	2010 [70]
Piraino 1, Sicily	coprolite/mummy	1	ABC	Callen/Lyco	2010 [48]
El-Deir, Oasis of Kharga, Egypt	coprolites and sediments from mummies	12	A	Reims	2010 [54]
**Dangjin, South Korea**	coprolite/ mummy	1	AB	Lutz	2011 [39]
**Gongju, South Korea**	coprolite/ mummy	1	AB	Lutz	2011 [39]
Antelope Cave, AZ, USA	coprolites	20	ABCD	Callen/Lyco	2011 [29]
**Sapgyo, South Korea**	coprolite/ mummy	1	AB	Lutz	2012 [39]
CMC, Mexico	coprolites	36	ABCD	Callen/Lyco	2012 [33]
Zweeloo, Belgium	mummy intestine section	1	ABC	Searcey	2013 [56]
**Jinju, South Korea**	coprolite/ mummy	1	AB	Lutz	2014 [39]
**Hadong-2, South Korea**	coprolite/ mummy	1	AB	Lutz	2014 [39]
**Sacheon, South Korea**	coprolite/ mummy	1	AB	Lutz	2014 [39]
**Mungyeong, South Korea**	coprolite/ mummy	1	AB	Lutz	2014 [39]
**PJ-SM, South Korea**	coprolite/ mummy	1	AB	Lutz	2014 [39]
Vilnius, Lithuania	mummy intestine sections	10	AC	Searcey	2014 [49]
Nivelles, Belgium	Burials coprolites and Sediments	3	AC	HCl	2015 [34]
Furna do Estrago, Brazil	coprolites/burials	6	AC	Lutz/Lyco	2015 [28]
Mamluk Period, Jerusalem	coprolites	12	AC	Reims	2015 [55]
CMC, Mexico	coprolites	100	ABCD	Callen/Lyco	2017 [26]

^a^*n*, number of samples analyzed

^b^“Type” refers to whether the derived data are reliable for positive/negative (A), prevalence (B), infection intensity (C), or overdispersion (D) studies

*Abbreviations*: HCl, studies that needed to use acidified rehydration solution following [59]; Lyco, analysis included epg estimation by using *Lycopodium* spores

Coprolites and mummies are most common in the Americas and archaeologists have excavated them since the 1940s. Large numbers of coprolites were analyzed beginning in the 1960s. Therefore, these data are dominated by North and South American studies. Korea’s Joseon Dynasty mummies are presented in bold, since are part of other geographic area and context

The last two decades of the 20th century were a time of geographic expansion of study areas and exploration of cultural influences on parasitism. As reviewed by Araújo and colleagues [8], Brazilian work emerged in the nineteen eighties. Discoveries in Brazil and Chile led to papers on the origins and dispersal of common parasites as well as the first papers on animal coprolites. In North America, the influence of cultural trends was defined and parasitological data were related to bone pathology data, especially porotic hyperostosis [1–3, 9].

The first decade of the 21st century was a time of review and consolidation of findings. A volume of the *Memórias do Instituto Oswaldo Cruz* was dedicated to “paleoparasitology”. Thirty articles were presented covering new methods, new theories, case studies and summaries of findings. Two new perspectives were introduced. The pathoecology perspective was introduced by Martinson and colleagues [10]. She presented a case study that united archaeological reconstruction of cultural patterns and life-cycles of parasites to define risk factors of infection and pathology for villages in Peru. Eggs per gram values were introduced in this study to quantify infection levels for different sites. Reinhard & Buikstra [11], introduced quantification of louse infestation to determine whether or not the negative binomial distribution of overdispersion could be seen in archaeological data. Both of these perspectives were developed in subsequent years and the overdispersion concept, when combined with EPG estimates, can be a powerful approach to determining infection intensity.

Pathoecology allows for the generation of testable hypotheses based on archaeological data and knowledge of parasite life-cycles [1, 3, 12–14]. The field is based on Pavlovsky’s nidus concept [15] applied to archaeology [16]. Reinhard & Bryant [3] wrote, “The nidus is a geographic or other special area containing pathogens, vectors, reservoir hosts, and recipient hosts that can be used to predict infections based on one’s knowledge of ecological factors related to infection. Ecological factors include the presence of vectors, reservoir hosts, humans, and external environment favorable for the transmission of parasites. An individual nidus therefore reflects the limits of transmission of a given parasite or pathogen within specific areas of interaction: bedbugs in a bedroom, for example. Thus, a nidus is a focus of infection. A nidus can be as confined as a single room containing a bed and with access to the room by rodents carrying plague-infected fleas. However, a nidus can also be as large as the community and its surrounding area in which there is a transmission of hookworms.”

Reinhard & Araújo [14] refined the concept to develop a predictive tool that in turn can be used to develop hypotheses testable via archaeological investigations. For the Lower Pecos Canyonlands, they assimilated the distribution of natural definitive hosts with an overlay of the distribution of intermediate hosts, and integrated the distribution of hunter-gatherer features that would have expanded the nidi for infection. Based on this work, the authors recommended excavation and laboratory strategies to recover evidence of parasite transmission.

Archaeological data can have practical value to modern epidemiology. The continuity from archaeological to modern patterns has been shown with mummies and coprolites from Asia. Han and colleagues [17] reviewed studies from Korea and China and showed that comparisons between modern and ancient parasite data have been done for some time. Korean colleagues opened this current trend in diachronic epidemiological study comparing infection prevalence and distribution between late 20th century parasite infection surveys and evidence from the Joseon Dynasty of the 1400s to 1800s in South Korea [18, 19]. The distribution of *Trichuris trichiura* and *Ascaris lumbricoides* were the same between the two periods. However, the prevalence of trematode species was higher for the Joseon people and some flukes had a broader distribution in the Joseon times relative to modern times. Hookworm emerged after the Joseon Dynasty. These studies showed that if methods are consistently applied, then data coming from archaeological contexts are comparable to modern clinical contexts.

In the last decade, quantification methods were applied to coprolites and mummies to estimate EPG values. This was a methodological breakthrough that opened the possibility of estimating infection intensity and relative pathological implications. Furthermore, this allows parasitologists to examine overdispersion in archaeological populations. These methods allow us to recover parasite data that can be examined from an epidemiological perspective.

**Fig. 1:**
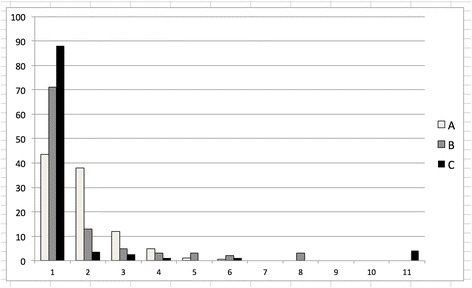
Graph derived from three examples of endoparasite overdispersion [23, 24]. Example A, a more marginal example of aggregation shows data for tapeworm infection (*Triaenophorus nodulosus*) in perch (*Perca fluviatilis*). In this example 54% of the tapeworms were in 18.5% of hosts with 81.5% uninfected or lightly infected. Example B shows pronounced overdispersion of the nematode (*Porrocaecum ensicaudatum*) in starlings (*Sturnus vulgaris*). In this case, 89% of the hosts are uninfected or lightly infected, and 69% of the parasites were recorded in 11% of the hosts. Example C shows a very pronounced case of overdispersion for nematodes (*Spiroxys japonica*) in pond frogs (*Rana nigromaculata*). In this case, 70% of the parasites were recorded in just 4% of the hosts while 88% of the hosts were uninfected and 8% had light infections.

In the negative binomial distribution, the variance is greater than the mean, so the variance divided by the mean is greater than 1. Whenever the variance/mean is greater than 1, we say that the distribution is overdispersed or aggregated. Overdispersion characterizes a phenomenon of aggregation of a majority of parasites in a minority of the host population. Thus, the majority of hosts have no or few parasites. A very small number of hosts, however, carry a great number of parasites. Crofton [20] showed that overdispersion was present for parasite populations. Since then, overdispersion has been defined as axiomatic among parasites of a variety of vertebrate and invertebrate hosts [21–23]. Patterns of overdispersion from wildlife parasitology are presented in Fig. 1 derived from overview studies [23, 24]. This illustrates the pattern across species. Example A shows data for tapeworm (*Triaenophorus nodulosus*) infections in perch (*Perca fluviatilis*). In this example, the aggregation is not as pronounced as in other cases; 54% of the tapeworms were in 18.5% of hosts with 81.5% of hosts remaining uninfected or lightly infected. Example B shows data for nematode (*Porrocaecum ensicaudatum*) infections in starlings (*Sturnus vulgaris*). In this case, 89% of the hosts are uninfected or lightly infected, and 69% of the parasites were recorded in 11% of the hosts. Example C shows data for nematode (*Spiroxys japonica*) infections in pond frogs (*Rana nigromaculata*). In this case, 70% of the parasites were recorded in just 4% of the hosts while 88% of the hosts were uninfected and 8% had light infections. Overdispersion was discovered for four species of the most common human-infecting geohelminths [25].

**Fig. 2:**
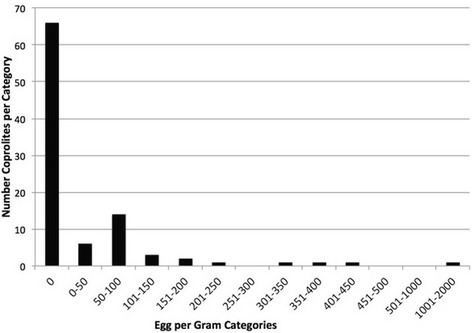
Graph derived on pinworm eggs recovered from La Cueva de los Muertos Chiquitos coprolites [26]. The graph exemplifies pronounced overdispersion with 66 of 100 samples negative. The ten samples with the highest counts contained 76% of the eggs. This is most similar to example C in Fig. 1

**Fig. 3:**
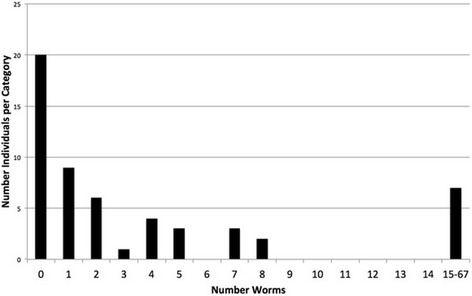
Graph derived from pinworm infection data from Korean school children [27] (Fig. 1, trial 2). The data were collected in several anthelmintic “dewormings”. One of three treatments revealed a dispersed, negative binomial distribution: 72% of the worms were recovered in 13% of the subjects while 53% were uninfected

To demonstrate normal versus overdispersed patterns in archaeological and modern humans, we compared data from coprolites excavated at La Cueva de los Muertos Chiquitos (CMC) (Fig. 2) with pinworm (*Enterobius vermicularis*) overdispersion in a clinical study (Fig. 3). The CMC data are drawn from Morrow & Reinhard [26] and the clinical data from Chai and colleagues [27]. The difference is that the Korean study was based on the recovery of worms from children compared to the CMC analysis, which was based on EPG counts from a diversified sample of coprolites. The Korean worm counts corresponded with a negative binomial distribution and 72% of the worms were recorded in 13% of the subjects while 53% were uninfected [27]. The CMC data are also overdispersed with 66% of samples being negative for pinworms. The ten samples with the highest EPG counts contained 76% of the eggs. This is of interest from two perspectives. First, the CMC data show aggregation in that a minority of coprolites contained a majority of the eggs. The second point is that overdispersion in pinworm egg counts is not necessarily intuitive in context of the pinworm life-cycle. Pinworms lay substantial numbers of eggs on the perianal folds. Therefore, one might not expect to find eggs within coprolites. However, the CMC data indicate that EPG concentrations for pinworms can be used to document overdispersion.

To paleopathologists, overdispersion of parasites is important from several perspectives. First, infected hosts exhibit lower fitness. This might be signaled in highly infected hosts by lower fecundity, slower growth rates, more severe expression of pathology or higher mortality rates. In the paleopathological record, the osseous evidence of short stature, non-specific stress indicators, and skewed age-at-death ratios could well be the influence of overdispersion on individual fitness. A second relevant issue relates to parasite transmission by heavily infected individuals, sometimes called “superspreaders”. In modern infection control strategies, superspreaders are targeted for infection management. Simply put, clinical examination diagnoses the superspreaders who are treated and subsequently the transmission of the parasites is reduced. Paleopathologically, evidence of treatment of heavily infected individuals has been found as reviewed by Teixeira-Santos and colleagues [28]. In some cases, evidence of medicinal plants is associated with highly infected individuals [29]. Thus, the connection between infection, spreaders and treatment was recognized in prehistory.

In the broader picture, overdispersion relates to regulation of host populations since aggregation of parasites is a stabilizing force in host population dynamics. In bioarchaeology, relative population success, as represented by pathology such as porotic hyperostosis, may be a result of disruptions of life styles by environmental change, crowding, or other issues [3, 12, 30, 31]. In summary, we can make these connections from modern epidemiology to past epidemiology through the analysis of overdispersion. Aggregation of parasites among hosts is important because individual-level parasite loads determine individual host fitness and transmission potential. Paleopathologically, the individual-level conditions amplify on a population level to influence transmission and then the bioarchaeological stability of populations through time.

Bryant & Reinhard [30] referred to coprolites as the “missing links” in paleopathology. Coprolites contain the data needed to define diet and infection. These were two essential factors that defined over-all health in prehistory. Infection prevalence and intensity can be documented between sites with new EPG quantification methods. However, fewer quantified data sets have been collected during the past decades. We believe that these data can address three issues. First, at the level of individuals, good diagnoses of any sample(s) can be used to trace parasites through time and space, which are relevant to paleopathologists. Secondly, data from large, diverse samples can be used to assess prevalence, which are relevant to understanding pathology at a population level. These population data then become relevant to bioarchaeologists. Thirdly, defining EPG leads to estimating infection intensity, and ultimately overdispersion. These topics are especially powerful for assessing disease in the past at both the individual and population levels, which is relevant to paleopathology. At this early state, EPG methods are currently being developed in independent laboratories utilizing different methods. Herein, we assess the ease and comparability of these methods and discuss these important issues in developing an approach to studying parasite overdispersion among those who occupied archaeological sites.

It must be noted that clinical methods were tested and reviewed in several laboratories in the 1960s-1980s [32]. The combined experience showed that with coprolites and mummies, clinical techniques were not consistently successful. Continued research in the last decades came to the same conclusions [33–35]. Also, for most studies of coprolites and mummies, detailed analyses of diet were required [3, 30]. Therefore, methods had to be developed to recover seeds, pollen, starch grains, and parasite eggs. This goal was accomplished during the 1960s (see reviews by Reinhard & Bryant [3] and Bryant & Reinhard [30]).

This paper focuses on methods developed for coprolites and mummies. By reviewing the development of methods used on these remains, we will define which data sets collected over the last six decades are applicable to studies of distribution, prevalence and quantified epidemiology. Preservation conditions for coprolites and mummies are best in the Americas. A majority of the European parasite work in historical or archaeological material has centered on latrines, which are not amenable to overdispersion studies, as they contain combined refuse from an unknown number of individuals, often spanning several temporal horizons. Therefore, this paper has a mostly American focus.

## Development of methods

The goal of sampling should be to derive samples from a diverse set of individuals. Ideally, one would have some independent measure of the number of people occupying that site (e.g. burials), but in most cases that is not easily possible. Corroborative archaeological evidence is sometimes available. For example, Morrow & Reinhard [36] used an analysis of dental casts made from quids to infer that a population of at least 50 people contributed to the sample based on distinct dental cast morphology [37]. Archaeological estimates of population size based on room numbers has also been used [12]. For each site, the parasitologist should obtain at least an estimate of the size of population that used the site and over what time range. Then he/she can develop a sampling strategy that as close as possible statistically determines how many samples are needed to get an informative representation of prevalence. For some sites, an appropriate sample number can simply not be met, because there are only so many preserved coprolites. In such cases, it is especially important to report the effect on the confidence level of the statistical inference as recommended by Jovani & Tella [38].

A guide for sample size selection should be based on modern studies of prevalence estimates at different sample sizes of actual populational prevalence figures. Jovani & Tella [38] completed an extensive review of prevalence studies. For minimum sample sizes between 10 and 20, they recommended using 15 as a reasonable trade-off for maintaining acceptable levels of uncertainty. At sample sizes ranging between 20–100, reliable prevalence data are obtainable. Therefore, for prevalence data, sample sizes of 16 or greater should be used [38]. The reliability of the data increases with larger sample sizes and researchers must acknowledge the influence that sample size has on their conclusions. Also, this is only relevant for reasonably high prevalence levels. If prevalence levels are very low, large sample sizes are needed in any case for detection. This is a very important consideration for archaeological parasitologists. For some archaeological cultures, high prevalence did occur [16, 18, 39]. However, prevalence was very low among hunter-gatherers across the western arid regions of North America [7, 9]. Reinhard & Bryant [40] (p. 245–288) and Reinhard [9] asserted that many samples, as close to 100 as possible, are needed for parasite prevalence studies for hunter-gatherers. The prevalence of parasite infection for hunter-gatherers ranged between 0–4%. Therefore, sample sizes of 50 to over 100 were used to ensure that evidence of true infections was found. However, when these methods were applied in the 1980s and 1990s to agricultural sites, it became apparent that sample size could be reduced because parasite prevalence increased to 3–29% among agricultural people. Because it takes a tremendous commitment of time and trained personnel to conduct parallel dietary analyses, it became attractive to researchers interested in the parasite-diet interface to be able to reduce sample size to 30–50 coprolites while maintaining rigor in data collection.

Based on the discussion above, one might think that higher sample size is always better. This is not necessarily the case. From the archaeological perspective, on must also consider diversifying provenience sampling. If high number samples are based on sampling fewer proveniences, then the sample will be skewed by individuals represented in limited contexts. Therefore, archaeological samples must consider both number of samples and number of proveniences from each site. It is often necessary to reduce the number of samples for sites that have limited distinct proveniences.

Sample size and sample diversification are essential in assessing overdispersion and prevalence. It is important to have large numbers of samples and for these samples to come from diverse archaeological contexts. Secure sample diversification is achievable when samples are recovered from burials or mummies because each sample is associated with a specific person. Reinhard [41] addressed methods for non-burial archaeological contexts. Different sites exhibit different sanitation strategies and some of these sites have many small latrine features. Diversification can be achieved by analyzing one coprolite from each individual feature. More often, archaeologists encounter large deposits containing hundreds to thousands of samples. In such cases, diversification can be achieved by focusing the sampling strategies on grid squares and levels [41]. Finally, at some sites, coprolites are recovered in discrete contexts each isolated from the other. These individual deposition episodes represent activities separated by significant time passage and every sample under these conditions can be included.

This conclusion is supported by examining modern prevalence assessments. Jovani & Tella [38] completed an extensive review of prevalence studies. They define low sample sizes as 1–15, at which there is poor prevalence statistical reliability. At sample sizes ranging between 16–100, reliable prevalence data are obtainable. Therefore, for prevalence data, sample sizes of 16 or greater should be used. However, the reliability of the data increases with larger sample sizes and researchers must acknowledge the influence that sample size has on their conclusions. In Table 1, we note studies with sample sizes larger than 15 as appropriate for prevalence assessments.

In archaeological samples of mummies from specific cultural horizons, aggregated prevalence data have been derived from individual studies of difference cemeteries. For example, 20 Joseon Dynasty mummies have been recovered and analyzed (Table 1). Because these are derived from the same cultural horizon and cultural class, they have been used to assess the prevalence of parasites specific to the higher socio-economic class of this culture [39]. This is, in our assessment, a reasonable approach to parasite prevalence assessment.

Sample size for overdispersion studies should follow the guidelines for prevalence analysis. A minimum size of 16 samples should be used for these examinations. However, we are experimenting with larger sample sizes (50–100) to obtain data for the common parasites as well as for more rare infections [26, 42].

There are further archaeological considerations relevant to interpreting even large and diverse samples. The power of inference depends on the geographic scale considered. Even if the prevalence is derived from a good and statistically relevant sampling from a single archeological site, it may not reflect the prevalence of a large population dispersed over multiple sites within a limited geographic space. This had been recognized in some research areas for decades leading to multiple Ancestral Pueblo site studies [7]. The benefit of multiple analyses over time was ever increasing fine-grained results delimiting parasitism patterns for sub-regions and time periods [9]. This is also because - as noted in the nidus concept above - infected individuals are not necessarily evenly distributed among the population, but may occur “clumped”, in certain favorable ecologies, but not in others [12]. Or in other words, several nidi were involved in the manifestation of overdispersion on a landscape level. This extended to the diversity of parasites across space and time. Because of the relative abundance of Ancestral Pueblo sites, fine-grained ecological comparisons were possible. However, for archaeological populations that left a more sparse record, and especially hunter-gatherers, we must keep in mind that any sample may only represent a seasonal, transitory parasitological snapshot. The data from each sample must result in a more circumspect interpretation. In general archaeoparasitologists recognize that samples come from a stratified landscape of prevalence across subpopulations, time, and often unknown topography.

Coprolite analysis began earnest in the 1960s (Table 1). Samuels [43] published a very early rehydration solution of NaOH and EDTA. By the 1970s, methods had been standardized by researchers in Canada, Peru and the USA. Although early researchers experimented with clinical methods of the day, such as formalin-ether separation and zinc sulphate (ZnSO_4_) flotation, the development of a simple rehydration method was rapidly adopted by institutions that hosted coprolite research.

### The Callen method

Canadian researchers Eric Callen and T. W. M. Cameron adapted the rehydration method of Van Cleave & Ross [44] as the standard first step in coprolite methods [45–47]. It is important to remember that Callen and Cameron were a botanist/parasitologist team that established the interdisciplinary approach to coprolite analysis that is used today [46]. Their interdisciplinary need to recover all types of data from samples defined the rehydration method subsequently applied by North American researchers. This method, henceforth called the Callen method, employs 0.5% trisodium phosphate (Na_3_PO_4_) aqueous solution to rehydrate coprolite samples. As applied today, this method facilitates the recovery of parasites, pollen, starch, and macroscopic dietary remains. Reinhard & Bryant [3] present a detailed literature review of subsequent articles, chapters, theses, and dissertations that were built on this method.

The Callen method, as applied from 1970 onwards, involves rehydration, disaggregation, and screening microscopic remains, followed by parasitological and dietary analyses. As applied today, the Callen method begins with describing, cleaning, photographing, and weighing each sample. The samples are subsampled, ideally by sectioning the sample longitudinally. For each sample, one portion is preserved for future analysis and the second portion is processed. The subsamples are weighed and rehydrated in 0.5% trisodium phosphate aqueous solution for 48 hours. After this time, the samples are transferred into beakers on stir plates and disaggregated using a stir bar. Disaggregation continues until the microscopic particles are completely separated from macroscopic fibers, bones and seeds. The disaggregated remains are poured through 250 μm mesh screens over a second series of beakers. Using distilled water jets from wash bottles, the macroscopic samples are washed while being separated with laboratory minispatulas and glass stir rods. In this way, the microscopic remains are completely removed from the macroscopic remains. Following screening, macroscopic remains are transferred from the mesh screens to sterile filter paper, labeled, and left to dry for analysis. The dried macroremains are later examined using dissection microscopes. The water and microscopic residues that pass through the screen and are collected in beakers are subsequently concentrated *via* repeated centrifugation. Microscopic examinations are conducted utilizing an aliquot of the microremains.

This basic Callen method has been modified in recent decades to permit EPG concentration [10, 29, 33, 34, 48–51]. At the end of the 48 hours rehydration period, one or more tablets of *Lycopodium* spores (available from the University of Lund, Sweden) are dissolved in 1–5% HCl and added to the rehydrating coprolites. In general, one tablet is added per gram of sample. These spores mix with the samples during the disaggregation phase. The concentration of EPG can then be estimated by dividing the number of eggs counted by the number of *Lycopodium* spores counted. This quotient is multiplied by the number of spores added and then divided by the weight of the subsample.

### The Lutz method

In Brazil, the Callen method was combined with the spontaneous sedimentation method [52]. This method is reviewed by Camacho and colleagues [53]. Samples are rehydrated in 0.5% trisodium phosphate aqueous solution (Na_3_PO_4_) for 72 hours [45]. After this period of time, the samples are disaggregated with a glass stir rod, strained through triple folded gauze on a glass funnel into conical glass jars and left to sediment for 24 hours. Drops of the sediment are taken from the bottom with Pasteur pipettes to make microscope preparations.

Korean researchers use a slightly modified spontaneous sedimentation method [19]. The samples are rehydrated in 0.5% trisodium phosphate aqueous solution over a week-long period. During this rehydration period, the samples are shaken several times to ensure disaggregation. After the rehydration, samples are filtered through several layers of gauze and precipitated for 1 day. Then, the precipitates are dissolved in 10% neutral buffered formalin, and pipetted onto microscopic slides.

Very recently, researchers experimented with *Lycopodium* quantification of EPG with the Lutz method [28]. A tablet of *Lycopodium* spores is dissolved in HCl and added to the rehydrating coprolites. This is followed by a thorough disaggregation to homogenized microscopic remains in the rehydration fluid and to separate them from larger food remains, such as bone fragments and seeds. After this, the material is strained in double gauze folded into conical receptacles where they were left to sediment for 24 hours.

### The Reims method

This method was formally named and defined by Le Bailly and colleagues [54]. The method was developed by Françoise Bouchet at the Université de Reims, France. Le Bailly and colleagues [54] modified the method by reducing sonication time. Samples are first rehydrated for 10 days in a 0.5% trisodium phosphate aqueous solution and 5% glycerinated water solution. Several drops of 10% formalin are added to prevent fungal or bacteria growth. The samples are then crushed using a mortar and pestle and subjected to ultrasonic treatment for 1 min, to mix the solution and separate parasite remains from the sediment. The solution is filtered in a column of sieves, with mesh sizes of 315, 160, 50 and 25μm, using an ultra-pure water constant flux system (Millipore, Direct-Q 5 system, Molsheim, France). The sediment retained by each sieve is stored in tubes with several drops of 10% formalin.

A modification of the Reims method was presented by Yeh and colleagues [55]. The specific modification was that 0.2 g of each coprolite was examined microscopically until the entire sample was analyzed. Afterward, the number of eggs counted was multiplied by 5 to determine the EPG.

### The Searcey method

Some mummies do not retain coprolites in the intestine sections. For such cases, an intestinal wash method has been developed [49, 56]. First, an intestinal segment is described, photographed, and weighed. The section is then placed in a gridded (1 cm^2^) Petri plate and rehydrated using 0.5% trisodium phosphate aqueous solution. During rehydration, the section increases in size and the grid helps to document this phenomenon. For a control sample, the exterior of the rehydrated intestine is then washed for microscopic remains. The rehydration fluid from the petri plate is poured through a 250 μm mesh screen covering a 600 ml glass beaker. The section is then placed in the screen and washed with a jet of distilled water while being gently scraped with a lab minispatula to loosen any adherent material. Following screening, macroscopic remains are transferred from the mesh screens onto sterile filter paper, labeled and left to dry. The dried macroremains, if recovered, are later examined *via* stereomicroscopy. The microscopic remains in the 600 ml beaker are concentrated *via* repeated centrifugation and serve as analysis control.

The section is transferred to a fresh Petri plate. Then it is opened along the longest dimension with a scalpel and the section is unrolled. The interior of the section is washed with a jet of distilled water through a 250 μm mesh screen covering a 600 ml glass beaker. The fluid in the beaker is concentrated via repeated centrifugation. Following screening, macroscopic remains are transferred from the mesh screens onto sterile filter paper, labeled and left to dry. The dried macroremains, if recovered, are later examined *via* stereomicroscopy.

Two centrifuge tubes of microscopic remains result from this process and should be labeled “interior” and “exterior control”. The number of milliliters in each sample is recorded. A *Lycopodium* spore tablet is dissolved in HCl and added to each tube. The microscopic remains are then washed several times in distilled water before microscopic analyses begin.

### Miscellaneous methods

Methods for analyzing mummies are adapted according to the conditions of preservation. As reviewed by Seo [39], the majority of Korean mummy studies is based on coprolites recovered from intestinal tracts. Therefore, the Callen or Lutz methods are applicable. However, for some South American mummies, trisodium phosphate does not rehydrate remains. In such cases, a 4% solution of potassium hydroxide has been successful (unpublished observations). Hidalgo-Argüello and colleagues [57] used 7% potassium hydroxide, combined with the clinical McMaster method, to recover eggs from mummy abdominal contents and other entombed remains.

### Skeletal analysis

Coprolites are sometimes recovered from skeletons [34, 58] or open-air sites [59]. Often, such remains are calcified and for this reason the rehydration with 0.5% trisodium phosphate aqueous solution is not successful. However, such samples rapidly disaggregate in 4% HCl solution and can be processed as described for the Callen method. In Table 1, this method is signified by “HCl”.

### Comparability

The methods are summarized Table 2. It is important to note that the labs from the (i) Escola Nacional de Saúde Pública, FIOCRUZ, Brazil, (ii) Laboratorio de Zoonosis Parasitarias, Departamento de Biología, Universidad Nacional de Mar del Plata, Argentina and (iii) Anthropology and Paleopathology Laboratory, Institute of Forensic Medicine, Seoul National University College of Medicine, Seoul, Korea, share samples with the (iv) Pathoecology and Palynology Laboratory, School of Natural Resources, University of Nebraska-Lincoln. Through this interchange, we have learned that the Lutz method as applied in Argentina, Korea and Brazil produces comparable prevalence results with the Callen method [33]. However, the gauze used in the process is not conducive to the total separation and concentration of microscopic remains *via* centrifugation as employed in the Callen method.

**Table 2:** Comparison of major coprolite and intestinal wash methods.

Method	Prelim^a^	Quant^b^	Rehydrat^c^	Disaggreg^d^	Screening^e^	Concent^f^	Post-analysis^g^
Callen	Cleaned, imaged, sectioned or cored	Lyco	0.5% Na_3_PO_4_; 48hr	Magnetic stirrer, active separation of particles w/ water jet and spatula	250 μm mesh	Centrifuge	Retain all macro and micro remains and unprocessed section
Lutz, Korea	–	–	0.5% Na_3_PO_4_; 1 wk	Agitation	Three layers of double gauze	Passive sediment 1 day then mixed w/10% neutral buffered formalin	–
Lutz, Brazil	Cleaned	–	0.5% Na_3_PO_4_	Glass stir rod	Three layers of double gauze	Passive sediment 1 day	Retain all macro and micro remains and unprocessed section
Reims	–	–	0.5% Na_3_PO_4_ in 5% glycerin- water w/ formalin	Crushed then ultrasonic treatment	Screened w/315 mm, 160 mm, 50 mm, and 25 mm meshes. Micro remains retained on screen		–

^a^“Prelim” refers to preliminary preparation of samples

^b^“Quant” refers to egg per gram (epg) quantification method

^c^“Rehydrat” refers to solution and time

^d^“Disaggreg” shows how the rehydrated samples are disaggregated

^e^“Screening” refers to how macroscopic remains are separated from microscopic

^f^“Concent” refers to methods of concentrating microscopic remains

^g^“Post analysis” relates to sample conservation

*Abbreviations*: “Lyco” refers to the application of quantification method based on *Lycopodium* counting

The Searcey method is not comparable with coprolites because the quantification of mummy material is based on the volume of microscopic remains recovered from the intestinal washes. We are currently applying this method to more intestinal sections and will eventually build a data set from mummies that will be comparable with one another.

In a recent paper, Dufour & Le Bailly [60] compared the Reims method with the Warnock & Reinhard [61] method for recovering eggs from sediments. Comparison showed that the Reims method was deficient in eggs recovery. Judging from the graphs presented by Dufour & Le Bailly [60], about 52% of ascarid eggs and about 73% of trichurid eggs are lost in the Reims screening method. We recommend that researchers avoid the Reims method for coprolite analysis until it is further refined. Instead, we recommend the methods developed by Jones [59] for coprolites from open sites and applied by Rácz and colleagues [34] for burials as summarized above in Skeletal analysis.

Considering sampling estimation methods based in a population approach and coprolite processing, the criteria used for ancient parasite analysis is specified in Table 3.

**Table 3:** Criteria for rigorous quantitative paleoparasitological analyses aiming at a quantitative approach in coprolite studiesquantitative approach in coprolite studies

Sampling			Processing
Population size estimation per site	Diversification		The Callen method with *Lycopodium* spore quantification has been proven to be the best method for measuring eggs per gram
Number of rooms; corroborative archaeology; number of burials; number of documented dentitions [37]; modern prevalence studies or previous prevalence observations in the same site	Provenience		
	Mummies	Coprolites from latrines	
	Sample as many individuals as possible	Use archaeological strata (grid squares and levels) to devise a diverse sample	

## Geographic representation of the current data set

Table 1 shows that the samples processed by the Callen method number 1485. The number processed by the Lutz method amounts to over 100. This provides a large data set of comparable samples that has allowed researchers to look at parasite prevalence over time and space. These data have been used by past researchers to define prevalence of infection over large geographic areas [7, 62–67]. Examples of these prevalence studies are presented below.

The Great Basin is the largest geographic area of optimal preservation in the United States, taking up parts of California, Oregon and Idaho, half of Utah and nearly all of Nevada. As reviewed by Reinhard & Bryant [3], the area was the focus of intensive archaeological work and large numbers of coprolites were recovered from desert and lacustrine areas of the region. This allowed parasitologists to define the spread of parasites. In the desert regions of Utah and Oregon, tapeworm, thorny-headed worms, and pinworms infected hunter-gatherers for some 10,000 years [7, 29]. These parasites were absent in the lacustrine area of Nevada. However, fluke eggs were present in human coprolites. As of today, it is still unknown if these represent true infections of humans, or if the eggs were consumed with prey animals without causing human infections [66]. At the southern extremity of the Great Basin, but within the Great Basin cultural area, pinworms and thorny-headed worms have been found among agricultural peoples. The coprolite database from this area is robust enough to emphatically show how parasitism emerged over 10,000 years of time and differentiated based on ecological and technological variation.

Among Ancestral Pueblo populations in the Southwest USA, the prevalence of parasite remains in coprolites varied profoundly. For the Ancestral Pueblo cultures of the Colorado Plateau, pinworm prevalence was especially variable. Analysis of housing style and location shows that stone-walled villages had the highest prevalence figures while small villages had the lowest. Large stone-walled villages unenclosed by caves had high variation. Researchers related this variation to limited air flow in caves which promoted airborne infection combined with the crowd effect of many people living in a concentrated cave environment [68]. Large villages outside of caves exhibit variance to differences in population size and patterns of space use, especially in terms of plaza and roof usage [12]. The ancestral pueblo parasite database, combined with skeletal pathology evidence, revealed patterns in prevalence of parasitism that varied with pathology resulting from vitamin B_12_ deficiency [9, 31]. These analyses show how fine-grained interpretations can be made from prevalence data combined with archaeological reconstructions.

## Working towards a paleoepidemiological approach

Epidemiology was applied to archaeology in a series of papers published from 2003 and onwards. All were based on new quantification methods. Reinhard & Buikstra [11] quantified lice on Peruvian mummies and demonstrated that the negative binomial of parasite aggregation was evident in archaeological sources. This axiom in parasitology simply states that the majority of parasites of a single species will be concentrated in a few number of individual hosts, around 10%. This in itself raised the possibility that studies of large samples could reveal variance in overdispersion and intensity based on host population factors. Arriaza and colleagues [69] continued this approach to louse parasitism in large populations studies of mummies, which led to conclusions regarding prehistoric social interaction over time.

A series of endoparasite papers has emerged recently and most utilize *Lycopodium* quantification. Arriaza and colleagues [70] connected Chinchorro prevalence of fish tapeworm prevalence in mummies to El Niño events. Importantly, these researchers built their database with data derived from both Lutz and Callen methods. Martinson and colleagues [10] showed that variation in parasite infection occurred between villages in the same river valley. Santoro and colleagues [50] looked at Inca Empire expansion, which impacted the Lluta Valley of northern Chile. Prior to the Inca, farms were small communities dispersed in the valley. The Inca established a large central town for the farmers and due to taxes on maize, the farmers expanded their subsistence by including fish on their diet. Tapeworm infection became common with this dietary expansion. In addition, the crowd parasite, human pinworm, became established in the town. Before the Inca, this parasite had been absent in the valley.

The variety of these examples shows how the accumulation of the data presented in Table 1 has already been used in diverse studies. The next step was developing a new database based on quantification in terms of EPG. An obvious application of this is the determination of worm burden. It must be said that some researchers have published reservations about the direct connection of worm burden estimates from EPG calculations. Dainton [71] was the first to state concerns related to archaeological work. He pointed out factors such as parasite diurnal variation in egg production, variable distribution of eggs within the same fecal pellet, effects of differential moisture level between feces, and other concerns. However, more recent reviews substantiate the value of assessing *Ascaris lumbricoides* worm burden and estimating pathology based on EPG fecal counts [72]. An important observation presented by these authors is that the negative binomial distribution in EPG is reflected cross-culturally and in very different geographic areas [72]. When EPG calculations are related to pathology, these values have been determined: 1–1999 EPG = light infection; 2000–3999 = moderate infection; > 4000 = heavy infection. However, assessing worm number based on EPG might be confounded by the fact that *A. lumbricoides* individual fecundity is inversely related to increasing worm burden. Therefore, the higher the number of worms, the lower the egg production of each individual female. Kotze & Kopp [73] reviewed the evidence of density-dependent fecundity for other parasites and find that hookworms and perhaps whipworms also exhibit this trait. For this reason, paleopathological estimates of worm burden are presented as ranges of worms present in the host, or the average daily egg production of females per gram of sample. For example, using the average egg count of 14,000 eggs per female per day of infection with whipworms [74], a gram of coprolite that contains 50,000 EPG could be said to contain the product of 3.57 females. Multiplying this value by the weight of the entire coprolite sample will provide and estimate of the worm production per coprolite. Only if the entire contents of the colon are represented from a mummy or skeleton, is it possible to estimate the range of adult worms in the human host [34].

A simple example of the Callen *Lycopodium* method of EPG concentrations comes from the comparison of whipworm EPG concentrations from the published literature for Inca [50], Chiribaya [10], Rio Zape [33], Piraino 1 [48], Mamluk [55], Zweeloo [56], Vilnius [49] and Medieval remains [34, 59]. These EPG values are presented in Table 4. Regarding the highest count from the Lloyds Bank Pavement coprolite from Medieval York, England, Jones’s high counts of whipworm and maw-worm led him to conclude that the individual “was parasitized by at least a small number of maw-worms and several hundred whipworms. Such an infestation today would be classed as a heavy one, although well within the limits of human tolerance” [59]. Rácz and colleagues [34] came to a more dismal conclusion from their analysis of material from a Medieval skeleton recovered in Nivelles, Belgium. The Nivelles skeleton retained an intestinal tract represented by eight recovered coprolites. They calculated an average value of 51,630 EPG for the coprolites. This value, when multiplied by the weights of all the samples, yielded a total value of 1,500,000 eggs in all of the samples. They concluded that this represented a worm burden beyond human tolerance and likely contributed to the death of the individual. Kumm & colleagues [48] analyzed Piraino 1, which yielded a value of 34,529 EPG. This relatively high value likely resulted from the lowered immunity of Piraino 1, who died of metastatic cancer [48]. The remaining values in Table 4 show counts consistent with subclinical infections that provoked no symptoms. This simple analysis of a small sample of cases shows that egg quantification is essential for assessing pathology caused by parasite infection.

**Table 4:** Whipworm egg per gram counts from various sites. The egg per gram data are converted to average output of a female whipworm of 14,000 eggs per day [74]

Site	EPG/worm(s) per gram	Year [Reference]
Lloyds Bank Pavement, York	66,000/4.7	1983 [59]
Nivelles, Belgium	51,630/3.7	2015 [34]
Piraino, Sicily	34,529/2.5	2010 [48]
Inca, Arica, Chile	5400/0.4	2003 [50]
Chiribaya, Arica, Chile	1800/0.1	
Vilnius, Lithuania	4779/0.3	2014 [49]
Chiribaya, Ilo, Peru	2240/0.2	2003 [10]
Chiribaya, Ilo, Peru	435/0.03	
Zape Mexico	1127/0.1	2012 [33]
Mamluk Period cesspool	162/0.01	2015 [55]
Zweeloo, Netherlands	traces	2013 [56]

Other smaller studies demonstrated overdispersion in samples for human-specific, direct life-cycle parasites, such as pinworms. This trend was also evident in smaller samples from Andean coprolites. In reviewing the pinworm data from the analysis of Inca coprolites from Santoro and colleagues [50], overdispersion is evident. For the Inca study, 24 samples were examined and six were positive for pinworm. In positive samples, the EPG counts ranged from 700 to 2100. Sixty-nine percent of the eggs were found in three (12.5%) of the hosts. The mean intensity of infection was 1350 EPG. Pinworm females carry 4000 to 16,000 eggs when they are ready to oviposit. The Inca pinworm counts represent less than one worm’s egg production per gram of sample. Using Jones’s vernacular, these infections were “well within the limits of human tolerance” [59]. Can we expect to find overdispersion from multiple-host helminths? To answer this question, we are reviewing the fish tapeworm data recovered previously from analysis of Chiribaya coprolites [10, 50]. The fish tapeworm data from Chiribaya coprolites are intriguing. Previously, Martinson and colleagues [10] had identified a high prevalence of fish tapeworm infection among production class villages of farmers and fisherman. One aspect of the study was the analysis of 11 coprolites recovered from the site of Chiribaya Baja. Seven were positive for eggs and the numbers ranged from 90 to 17,800 EPG. Sixty-seven percent of the eggs were recovered from a single individual (9%) and 89% of the eggs were recovered from the two (18%) most infected individuals. These results are consistent with overdispersion. The mean intensity of infection is 3794 EPG which is a tiny fraction of the estimated daily egg production of a million eggs per day. Therefore, the high prevalence of 64% infection probably had little impact on health. This analysis of a small sample series demonstrates that overdispersion is present in archaeological helminth data and preliminary intensity data reflect variation. Thus, when quantification methods are applied to coprolites, comparable helminth analyses are possible.

To demonstrate aggregation in ectoparasites, we will use the louse data collected by Reinhard & Buikstra [11]. Lice data were collected from 146 mummies [11]. To quantify louse infestation, all nits and eggs were counted within a 2 × 2 cm area. Three counts were taken at the area of maximum scalp infestation and three for the area of minimal infestation. This was repeated for the hair three inches away from the scalp. Therefore, a total of 12 measurements were taken for each mummy. These data fit the negative binomial of overdispersion and heralded the emergence of true parasite epidemiology in mummy studies. Three sites were analyzed. The first was a large administrative center, Chiribaya Alta. The second village was El Yaral which specialized in Llama herding. The third site, Algodonal, was a small hamlet of refugees from the Lake Titicaca region who moved into the Chiribaya Alta area to escape the impacts of environmental collapse. Prevalence was variable between the sites: Chiribaya Alta (36%), El Yaral (18%) and Algodonal (71%). The mean intensity is a measurement of the average number of parasites for infested or infected hosts. For these sites, the mean intensity, as measured in terms of number of eggs/lice per cm^2^ varied: Chiribaya Alta (4.7), El Yaral (9.1) and Algodonal (12.4). The surprising contrast between the high El Yaral intensity and low prevalence is noteworthy. It shows that although fewer people were infested, those that were infested had heavy louse burdens. For the low status immigrants at Algodonal, the prevalence and intensity were both high. The lice data demonstrate that overdispersion is evident in archaeological data and that prevalence and intensity data are recoverable.

These small-sample examples show that quantification of helminth infection from coprolites is a promising area to explore. It is unfortunate that the truly large samples analyzed for parasites between 1970 and 1992 were processed before *Lycopodium* quantification was established [10, 50]. The analysis of such large samples would have allowed for the documentation of overdispersion and measures of infection intensity. Applying this type of work to large coprolite series associated with skeletal evidence of pathology could clarify the connection between parasitism, diet, and pathology. Dietary reconstruction is another avenue of coprolite research [30]. Previously, Reinhard [9] found a positive and significant correlation between pinworm prevalence in coprolites and cranial lesions of porotic hyperostosis in skeletons from the same sites and regions. These sites also differed in the number of parasites species evident in the samples. In addition, using the original Callen & Cameron [45] approach to coprolite analysis, while cooperating with bioarchaeologists, will allow researchers to explore both the diet and parasite factors that affected ancient health [31].

## Conclusions

The researchers focusing on archaeological parasitology had different goals through the years of study. The first works were focused on establishing the presence of parasites in ancient contexts. These pioneering studies defined the diversity of parasites in the Americas and Europe and developed methods for analysis. In the 1960s and 1970s, scholars began population-level studies that compared and contrasted parasitism in various geographic regions. At this time, prevalence studies dominated the field. Prevalence data stimulated interest in the consequences that parasite infections had among ancient people. This interest led to the question of whether infection provoked disease, which, in turn, led to investigations of paleoepidemiology. Paleoepidemiology required refinement of methods, especially quantification. This perspective necessitated the recovery of statistical data regarding overdispersion and infection intensity. Eventually, the paleoepidemiological approach will create comparable data from archaeological and modern human communities. For these reasons, quantification methods needed to be evaluated. The adaptation of *Lycopodium* spore quantification has been very successful when combined with the Callen method. However, it is not effective when combined with the Lutz method. This is due to the fact that it is difficult, or in some cases impossible, to mechanically separate the microscopic and macroscopic remains from the folds of gauze. We emphasize that the Callen-*Lycopodium* method is ideal for measuring EPG concentrations. Once EPG quantification is done globally, parasitologists working in archaeology will be able to clarify the conditions in which these people were living and associate infections to pathology. Interpreting the data within cultural and environmental contexts, the pathoecology of infections can be documented through time and space. In order to interpret ancient parasitological data on these perspectives, it is important to consider the quantification methods and also the concept of overdispersion on parasite-host systems within populations of the past. We hope that research on parasites in ancient material will continue in this direction and that the epidemiological perspective will be broadly applied to the interpretation of parasite infections among ancient populations.

## Abbreviations

EPG: Egg per gram
CMC: La Cueva de Los Muertos Chiquitos
NaOH: Sodium hydroxide
EDTA: Ethylenediaminetetraacetic acid
ZnSO_4_: Zinc sulphate
Na_3_PO_4_: Trisodium phosphate
HCl: Chloridric acid
